# Regional approaches for enhancing global health security

**DOI:** 10.1186/s12889-019-6789-y

**Published:** 2019-05-10

**Authors:** Rebecca Katz, Claire J. Standley

**Affiliations:** Center for Global Health Science and Security, Georgetown University, Washington DC, USA

Global health security represents the proactive and reactive efforts required to protect the world’s population from acute public health events. As is ever more evident in our interconnected world, a local threat can quickly become global in nature, threatening lives as well as economic and political stability. Many nations, however, lack the capacity to prevent, detect and respond to public health emergencies, although such core capacities are obligated under the 2005 International Health Regulations (IHR), and the importance of prioritizing this capacity building has been reinforced through multilateral initiatives such as the Global Health Security Agenda (GHSA).

Since entry into force of the International Health Regulations in 2007, most of the focus of global health capacity building has been at the national level. The requirements of the IHR, for example, apply to Member States; moreover, metrics for compliance have been largely based on demonstrating national capacities. Over the past decade, however, there has been increased recognition of the importance of supra-national or regional perspectives, particularly in terms of enhancing cross-border collaboration and identifying locally appropriate implementation strategies. In 2015, the World Health Assembly recognized the importance of regional exchange and coordination for sharing lessons learned related to achieving IHR compliance [1]. The WHO monitoring and evaluation tools for IHR also recognize the possibility for countries to leverage regional capacities in order to meet IHR requirements. For example, rather than require every country to establish a reference laboratory capable of all confirmatory diagnostic tests for national priority diseases, having an established relationship with a regional or international reference laboratory for confirmation of those pathogens is sufficient.

These changes showcase policy efforts to broaden the scope of global health capacity building; but what does implementation of regional approaches look like in practice? The WHO Regional Offices, operating with devolved responsibility from WHO headquarters in Geneva, have long integrated regional level planning with efforts to enhance health security. For example, the African Regional Office explicitly aligned its Integrated Disease Surveillance and Response framework, first developed in 1998, with IHR in 2010, with support from the U.S. Centers for Disease Control and Prevention (CDC) [2]. The World Health Assembly in 2017 also specifically called upon the six regional offices to develop regional operational plans for IHR implementation [3]. There are also numerous examples where supra-national geopolitical entities have been leveraged to establish organizations or agencies with a role in regional public health capacity building. For example, in 2011, the Caribbean Community (CARICOM) established the Caribbean Public Health Agency (CARPHA) in order to better align public health system strengthening efforts among member countries and establish a strong centralized agency under which regional challenges could be addressed [4].

External donors and providers of implementation assistance have also sought to approach global health security capacity building through a regional lens. For example, the World Bank’s Regional Disease Surveillance Systems Enhancement (REDISSE) project leverages the Economic Community of West African States (ECOWAS) and specifically its public health arm, the West African Health Organization (WAHO), to build surveillance, laboratory, workforce, and emergency response capacity across the region [5]. Technical assistance organizations can also directly establish partnerships within regions with a view to support multi-country efforts to prepare and respond to public health emergencies; this type of approach is exemplified by CDC’s Global Disease Detection (GDD) program.

For decades, the U.S. Centers for Disease Control and Prevention have been working with partners around the world to prevent, detect and respond to public health emergencies, including the last 10 years of the GDD program. GDD represents a highly effective model for dispersal of technical assistance and resources across more than one country, while also facilitating the host country to itself become a regional leader for promoting health security and improved health systems. From a donor perspective, the placement of regional centers can be used to complement and/or leverage existing regional entities and other biomedical and research assets in the same country. Overall, the scale of GDD’s impact is impressive, from the number of countries directly supported by GDD centers during outbreaks, to the discovery and characterization of dozens of new pathogens, and the professional training of hundreds of new field epidemiologists.

Of course, building health security capacities via regional centers comes with challenges. For example, it is difficult to develop the same depth of relationship across multiple countries, based out of a single regional office, as compared to establishing individual country offices. There may also be greater travel, personnel effort, and logistical costs associated with deploying staff from a central office to neighboring countries or further afield within a region, although concurrently regional offices may present cost savings through economies of scale and requiring a reduced overall footprint. Centralized nodes of concentrated technical expertise, that can be rapidly deployed around the region, may also align more closely with the agency’s mission, versus establishing smaller, more diffuse country-level representation that is required for longer-term development programs.

The challenges with building effective global health security capacity have been well documented. Since the revised IHR entered into force in 2007, countries have called on WHO and the broader international community to provide continued technical and financial assistance for implementation, while also rightly demanding that capacity building solutions are nonetheless tailored to local needs and systems. Regional models for technical assistance, such as that characterized by the GDD program and shown to be successful over the past decade of implementation, provide one such opportunity to strike this balance.
